# Foam Stabilization Process for Nano-Al_2_O_3_ and Its Effect on Mechanical Properties of Foamed Concrete

**DOI:** 10.3390/nano14181516

**Published:** 2024-09-18

**Authors:** Haibao Zhang, Zhenjun Wang, Ting Zhang, Zhaorui Li

**Affiliations:** 1 School of Materials Science and Engineering, Chang’an University, Xi’an 710061, China; haibaozhang@chd.edu.cn (H.Z.); zjwang@chd.edu.cn (Z.W.); lzr981013@163.com (Z.L.); 2 Xi’an Sipai New Materials Technology Co., Ltd., Xi’an 710061, China; 3 Shaanxi Union Research Center of University and Enterprise for Advanced Transportation Infrastructure Materials, Xi’an 710061, China

**Keywords:** foamed concrete, nano-Al_2_O_3_, foam stability, microstructure, mechanical property

## Abstract

Foamed concrete is increasingly utilized in engineering due to its light weight, excellent thermal insulation, fire resistance, etc. However, its low strength has always been the most crucial factor limiting its large-scale application. This study introduced an innovative method to enhance the strength of foamed concrete by using nano-Al_2_O_3_ (NA) as a foam stabilizer. NA was introduced into a foaming agent containing sodium dodecyl sulfate (SDS) and hydroxypropyl methylcellulose (HPMC) to prepare a highly stable foam. This approach significantly improved the foam stability and the strength of foamed concrete. Its drainage volume, settlement distance, microstructure, and stabilizing action were investigated, along with the strength, microstructure, and hydration products of foamed concrete. The presence of NA effectively reduced the drainage volume and settlement distance of the foam. NA is distributed at the gas–liquid interface and within the liquid film to play a hindering role, increasing the thickness of the liquid film, delaying the liquid discharge rate from the liquid film, and hindering bubble aggregation, thereby enhancing foam stability. Additionally, due to the stabilizing effect of NA on the foam, the precast foam forms a fine and uniform pore structure in the hardened foamed concrete. At 28 d, the compressive strength of FC0 (0% NAs in foam) is 2.18 MPa, while that of FC3 (0.18% NAs in foam) is 3.90 MPa, increased by 79%. The reason for this is that NA promotes the formation of AFt, and its secondary hydration leads to the continuous consumption of Ca(OH)_2_, resulting in a more complete hydration reaction. This study presents a novel method for significantly improving the performance of foamed concrete by incorporating NA.

## 1. Introduction

Foamed concrete is a porous and lightweight material created by adding foam, prepared through physical or chemical foaming methods, to a cementitious material paste [1]. Unlike ordinary concrete, foamed concrete from precast foam has a unique porous structure, which confers several advantages, including a light weight, thermal insulation, sound insulation, fireproofing, shock absorption, etc. [2,3,4]. These properties make foamed concrete widely used as a roadbed backfilling material and building thermal insulation material [5,6]. The properties of the foam prepared by foaming agents are crucial for the pore structure and performance of foamed concrete [7]. However, poor-stability foam leads to an inferior pore structure in foamed concrete, resulting in suboptimal strength and limiting its applications [8,9,10].

The performance of foamed concrete is often enhanced by improving the stability of the added foam [11]. Employing foam stabilizers helps mitigate the foam instability caused by inadequate foam dispersion and gravity drainage. There are two main types of foam stabilizers used in the early stage: one is cooperative foam stabilizers (e.g., lauryl alcohol [12], modified polyethoxylated silicone [13,14], etc.), which are able to enhance the interactions between the absorbed molecules on the surface, making the foam film more elastic and impermeable; the other type is viscosity-increasing foam stabilizers (e.g., xanthan gum [15,16], polyacrylamide [17,18], gelatin [19], carboxymethyl cellulose [20], etc.), which can increase the viscosity of the liquid phase, reducing the discharge rate of the foam and prolonging its half-life. However, the stability of the foam prepared using these methods is generally moderate [21].

Nanoparticle foam stabilizers (e.g., nano-SiO_2_ (NS) [22], nano-Al_2_O_3_ (NA) [23,24], nano-CaCO_3_ (NC) [2], wet-grinding fly ash [25], etc.) have been widely proposed for use due to their ability to create highly stable foam [26]. Nanoparticles can irreversibly and spontaneously absorb onto the gas–liquid interface, balancing the tension at the interface, forming solid–liquid–gas three-phase foams, preventing gas-phase transfer, and hindering physical drainage between the gas and liquid phases [23]. Additionally, the presence of appropriate nanoparticles can improve the strength of foamed concrete, especially NS and NA, due to their high hydration activity [22,27]. Zhang et al. [22] added amphiphilic NS to improve the mechanical properties of foamed concrete. It was found that amphiphilic nano silica changed the pore structure and improved the strength of the foamed concrete. Jiang et al. [2] studied the effect of NS and NC on the properties of foamed concrete; the result showed that its compressive strength was increased by 10% and 18% by adding NS and NC, respectively. Xiong et al. [24] demonstrated that incorporating NA enhanced the mechanical strength, drying shrinkage resistance, and uniformity of foamed concrete by increasing the strength of the cell walls through the pozzolanic reaction between NA and Ca(OH)_2_. Although the use of nanoparticles as foam stabilizers has often been reported to enhance the properties of foamed concrete, the existing research on the impact of NA on individual bubbles and the influence mechanism of NA-stabilized foam on foamed concrete is not sufficiently understood. Therefore, the influence and mechanism by which NA acts on foam need further study.

In order to improve the stability of foam and the strength of foamed concrete, high-stability foam has been prepared by adding NA to a foaming agent containing sodium dodecyl sulfate (SDS) and hydroxypropyl methylcellulose (HPMC) through physical foaming. The effects of different NA contents on the foam’s stability and microstructure have been investigated, and a mode of NA’s influence on foam stability has been proposed. Additionally, the impact of foams with varying NA contents on the strength, microstructure, and hydration products of foamed concrete has been examined. The results provide a novel method for improving the strength of foamed concrete from a novel perspective and demonstrate a basis for the application of NA in foamed concrete.

## 2. Materials and Methods

### 2.1. Raw Materials

In this study, Portland cement (P.O 42.5, purchased from Anhui Conch Cement Co., Ltd., Wuhu, China) conforming to the Chinese standard GB 175-2023 was used as the cementitious material, and its physical properties and main chemical components are given in Table 1 [28]. These chemical components were tested by an X-ray Fluorescence Spectrometer (XRF, Zetium, Malvern Panalytical), and the cement was dried in a 105 °C oven for 2 h before testing. SDS (obtained from Sinopharm Chemical Reagent Co., Ltd., Shanghai, China) was used as the foaming agent, while HPMC (obtained from Sinopharm Chemical Reagent Co., Ltd.) with a molecular weight of 200,000 g/mol and NA (obtained from Beijing Deke Daojin Science and Technology Co., Ltd., Beijing, China) with a particle size of 20 nm, were used as the foam stabilizers. Naphthalene sulfonate formaldehyde condensate (NSF, obtained from Linyi, China) with a solid content of 98% was used as the superplasticizer.

### 2.2. Preparation of Foams

**Figure 1:**
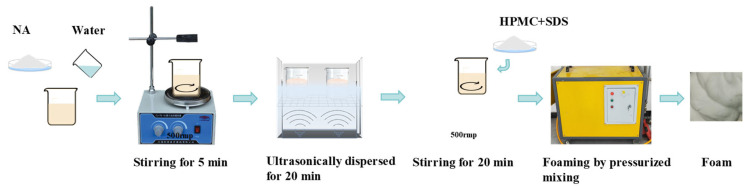
The foaming preparation process.

A high-pressure air foaming machine (LC-01B, input power: 3 kW, maximum pressure: 1.25 MPa) was used to prepare foam through an air compression method. Additionally, a CNC ultrasonic cleaner (KQ2200DB, voltage: 220 V, ultrasonic frequency: 40 kHz, ultrasonic input power: 100 W) was employed. The foaming preparation process and the mixed proportion of foaming agents are shown in Figure 1 and Table 2, respectively. First, NA was added to water and mixed by magnetic stirring for 5 min at 500 r/min, and then ultrasonically dispersed for 20 min to prevent the agglomeration of NA. Under magnetic stirring conditions, HPMC and SDS were added slowly and in sequence, and the foaming agent was obtained after magnetic stirring for 20 min. The prepared solution was then allowed to stand at room temperature for 20 min. Finally, the inlet of the foaming machine was then placed in the solution while ensuring the top of the inlet was completely submerged, and the foaming machine was turned on to prepare the foam.

### 2.3. Preparation of Foamed Concrete

To prepare foamed concretes with foams containing different NA contents (NA0, NA0.06, NA0.12, NA0.18, NA0.24, NA0.30, and NA0.36, respectively), 30 g of foam was added to a consistent proportion of cement paste (600 g of cement, 1.2 g of NSF, and a water/cement ratio of 0.45). The mixture proportions of the foamed concrete are shown in Table 3. The prepared foamed concretes were labeled as FC0, FC1, FC2, FC3, FC4, FC5, and FC6, respectively. The preparation process of foamed concrete was in accordance with the Chinese standard JGJ/T 341-2014 [29]. First, cement and NSF were mixed in a cement mixer (JJ-5) at a low speed for 60 s. Next, 2/3 of the calculated amount of water was added and mixing continued for 60 s. Then, the foam concrete slurry was prepared by adding the remaining amount of water and mixing for 300 s. Finally, fresh foam concrete was prepared by pouring the foam prepared by the foam machine into the slurry and mixing for 120 s at 120 r/min. First, cement and NSF were added to water and stirred for 150 s. Then, the foam produced by the foaming machine was added to the mixture and stirred for 120 s. The fresh foam concrete was then poured into 40 mm × 40 mm × 160 mm molds and covered with cling film. After 24 h of curing under standard curing conditions (20 ± 1 °C, relative humidity ≥ 900%), the molds were demolded and the specimens continued to be cured until the target ages (7 d and 28 d) were reached.

### 2.4. Experimental Methods

#### 2.4.1. Characterization of Foam

The foam’s stability was evaluated by the drainage volume and settlement distance of fresh foam, based on the Chinese standard JC/T 2199-2013 [30]. Fresh foam prepared by the foaming machine was immediately poured into a 500 mL measuring cylinder and excess foam was scraped off the top of the cylinder. The drainage volume (mL) and settlement distance (cm) of the foam were recorded at 20, 40, and 60 min, respectively. Each proportion of foam was tested three times for its drainage volume and settlement distance, and the average value was calculated.

An optical microscope (ISH500, produced by Shanghai Cewei Optoelectronic Technology Co., Ltd., Shanghai, China) was used to observe the morphology of the foam and to obtain the bubble size and liquid film thickness of the foam. The prepared foam was quickly placed on a glass slide and covered with a coverslip, leaving only one layer of foam. The entire sample preparation process was completed within 5 min.

Optical microscope photographs of the foam were processed using image processing software (ImagePy) to obtain the average diameter of the foam [31,32]. Each pixel point represents 31 μm.

#### 2.4.2. Characterization of Foamed Concrete

The flexural strength and compressive strength of the samples were tested using the Model TYE-300E Flexure and Compression Testing Machine (produced by Wuxi Jianyi Instrument & Machinery Co., Ltd., Wuxi, China) after curing for 7 d and 28 d, based on the Chinese standard JG/T 266-2011 [33]. The loading speeds were 50 N/s for flexural strength and 2 kN/s for compressive strength, respectively. The average value of three specimens from each group was taken as the result.

In order to observe and analyze the hydration products of foamed concrete, the specimens were broken into particles. The particles in the middle of the specimens were directly immersed in absolute ethanol for 7 d to stop hydration and then dried to constant weight in a vacuum oven at 50 °C. Small pieces 3–5 mm in size were selected for the SEM test. Some of the particles were ground into powder and sieved through a 63 μm sieve for the XRD test. The SEM (Hitachi S-4800, 10.0 kV) was used to observe the surface micromorphology of the samples. The XRD (D8 Advance, Cu-Ka radiation, scanning speed 0.5°/s, scanning step 0.02°, testing range 5°–60°) was used to characterize the physical phases of the samples.

## 3. Results and Discussion

### 3.1. Foam Stability

#### 3.1.1. Foam Drainage Volume

**Figure 2:**
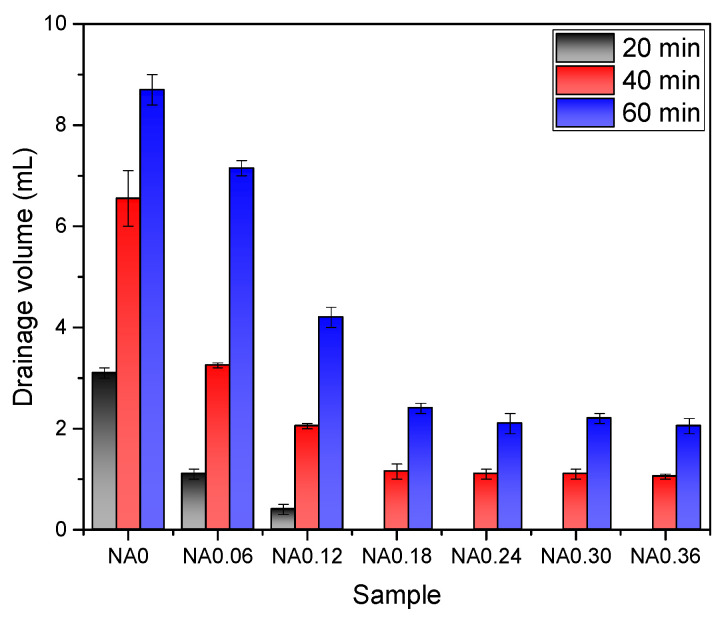
Drainage volume of foam with different NA contents after standing for 20 min, 40 min, and 60 min.

Owing to the force of gravity, liquid, when expelled, becomes concentrated at the base of the foam phase and flows through inter-bubble liquid channels, which is recognized as the gravitational drainage phenomenon. Figure 2 depicts the drainage volume of NA with time and at different concentrations to produce a drainage profile. The foam without NA drained more water, and its drainage volume was 3.1 mL, 6.6 mL, and 8.7 mL for 20 min, 40 min, and 60 min, respectively. The foam with added nanoparticles, on the other hand, showed different drainage phenomena, all of which were reduced at the same times. The addition of NA at a doping level of 0.18% reduced the drainage volume to 0 mL, 1.2 mL, and 2.4 mL at 20 min, 40 min, and 60 min. After that, the drainage volume does not change much with the increase in NA doping.

This indicates that the addition of NA has a delayed effect on the drainage of the foam, which significantly increases the drainage delay of the foam. Desbordes et al. [34] suggest that the initial drainage time is related to the plateau yield stress. When the yield stress exceeds the gravitational force of the draining fluid, the foam exhibits delayed drainage. As the bubble volume increases, the local yield stress decreases, and the bubble begins to drain when the yield stress is less than gravity. This suggests that the incorporation of NA molecules increases the local yield stress at the plateau boundary, thereby prolonging the time required for the local yield stress to decrease to the gravitational force of the draining fluid and achieving delayed drainage.

#### 3.1.2. Foam Settlement Distance

In addition to gravitational drainage, there is a destabilization mechanism in the form of bubble coarsening. Bubbles exert very high repulsive forces on each other. Foam roughening is due to the difference in pressure between two adjacent bubbles of different sizes. The gas pressure in the smaller bubble is greater than that in the larger bubble, so the gas in the smaller bubble has a tendency to diffuse into the larger bubble, resulting in aggregation and the loss of some of the bubbles. This phenomenon can lead to a decrease in the overall height of the foam.

**Figure 3:**
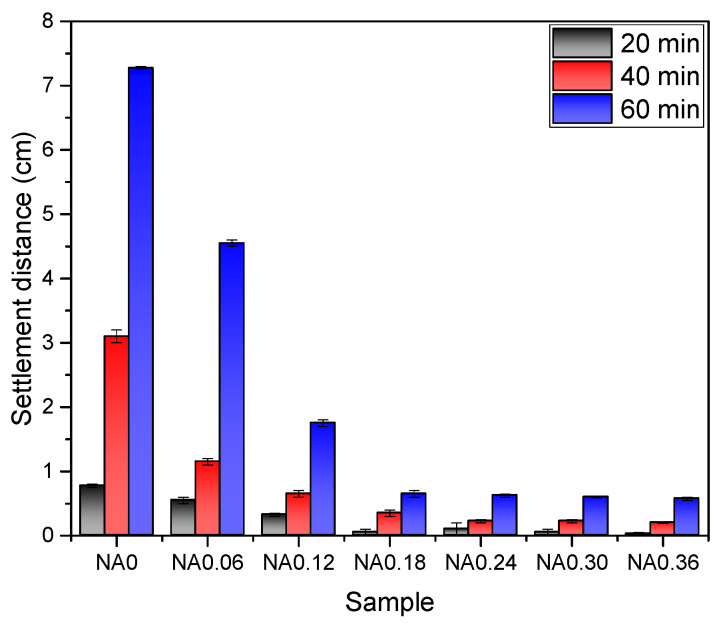
Settlement distance of foam with different NA contents after standing for 20 min, 40 min, and 60 min.

Figure 3 depicts the variation of the settlement distance of NA with time at different concentrations. More consistent with the change in drainage volume, the settlement distance decreased with increasing NA. The foam without NA had a larger settlement distance of 3.1 mL, 6.6 mL, and 8.7 mL at 20 min, 40 min, and 60 min, respectively. The addition of NA at a doping level of 0.18% reduced the settlement distance to 0.1 cm, 0.4 cm, and 0.7 cm when left for 20 min, 40 min, and 60 min.

Nanoparticles located within the gas–liquid–gas interface can generate a capillary pressure that helps to separate neighboring bubbles [35]. When bubbles try to merge, they have to overcome this capillary pressure, which undoubtedly increases the difficulty of bubble merging and thus weakens the tendency of bubbles to merge with each other.

From the test results of the drainage volume and settlement distance, it can be concluded that NA improves the stability of foam, which is the same as previous research has shown [23,24].

### 3.2. Microstructure of Foam

#### 3.2.1. Variation in Bubble Shape and Diameter Distribution

**Figure 4:**
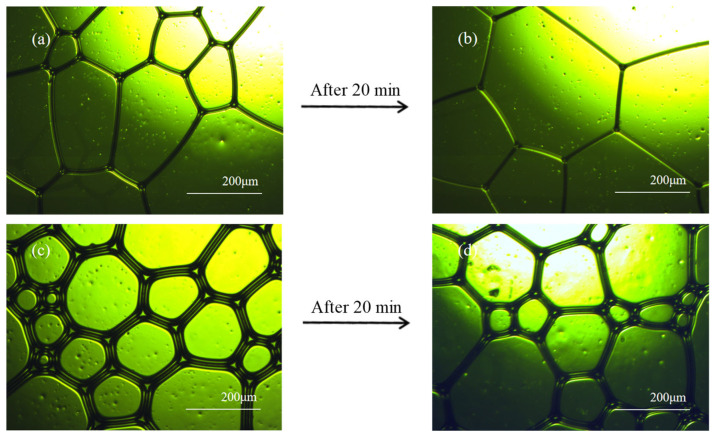
Foam morphology under optical microscope: (**a**) without NA and (**b**) without NA (after 20 min); (**c**) with NA and (**d**) with NA (20 min later).

Foam morphology changes were observed under an optical microscope for 20 min before and after the introduction of the foam stabilizing component, as shown in Figure 4. After adding the stabilizing components, the diameter of the foam bubbles decreased, their shape became more round, the liquid film became thicker, and the barrier between the foams became stronger. After 20 min, the foams without stabilizing components became larger in diameter, while those with NA only showed a slight enlargement.

**Figure 5:**
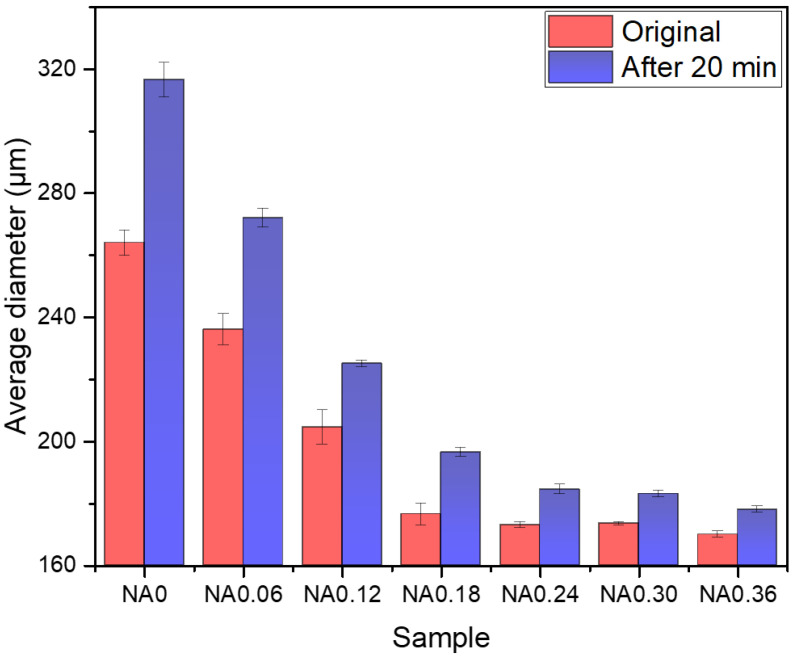
Effect of NA content on the average diameter of bubbles 20 min after their preparation.

Optical microscope pictures were processed to obtain the average bubble diameter using image processing software. As can be seen in Figure 5, the average bubble diameter decreases with the increase in NA. It can be seen that there is a tendency for the bubbles to increase in size with time, and this tendency also decreases with the increase in NA. The average diameter of the undoped NA bubbles was 264.4 μm initially and increased to 316.5 μm after 20 min, a difference of 52.1 μm. The minimum difference can be as little as 8.0 μm after NA doping, and the initial average diameter can be reduced to 170.1 μm.

#### 3.2.2. Mechanism of Foam Stability Improvement

**Figure 6:**
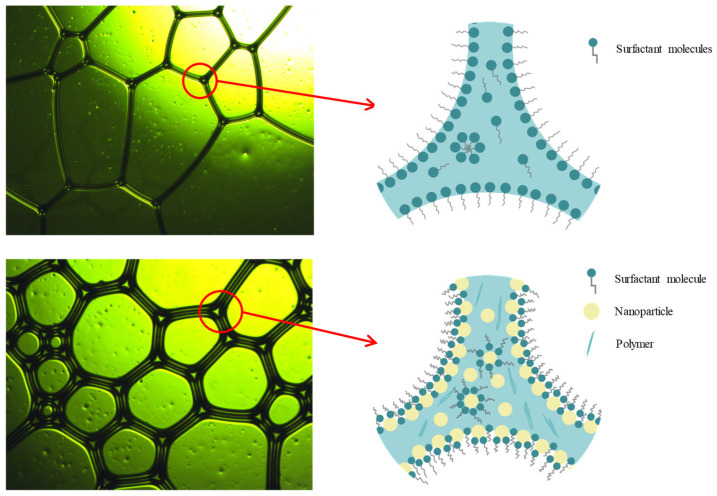
Mechanism diagram of NA’s ability to enhance bubble stability.

The mechanism diagram of NA enhancing bubble stability is shown in Figure 6. Surfactants have hydrophilic and hydrophobic groups at each end. With the addition of a surfactant, the surfactant reduces the surface tension by forming a film on the surface of the liquid and changing the molecular arrangement on the surface of the liquid. Surfactant adsorption at the gas–liquid interface also constitutes a protective barrier for the bubbles, which hinders the aggregation between the bubbles to a certain extent, but this effect is limited [36].

NA can also be adsorbed at the gas–liquid interface. The difference, however, is the NA requires much more energy than surfactant molecules during its desorption from the interface. This means that the NA is more tightly bound to the liquid film, and, therefore, the liquid film formed is more stable [37,38]. At the same time, some of the NA is also distributed between the bubbles, which can generate a kind of capillary pressure. This pressure helps to separate neighboring bubbles. When bubbles attempt to merge, they must overcome this capillary pressure, which undoubtedly makes it more difficult for the bubbles to merge, thus diminishing the tendency of the bubbles to merge with each other. In addition, the drainage phenomenon is mainly due to the flow of liquid in the bubble wall under gravity. NA is also irregularly distributed in the liquid in the bubble wall, which acts as an obstruction to the liquid’s flow.

### 3.3. Effects on Foamed Concrete

#### 3.3.1. Mechanical Properties

Foamed concrete is a porous, lightweight material composed of a matrix and pores, so its matrix properties and pore structure determine the properties of foamed concrete. Foamed concrete with a uniform pore distribution has better strength, and irregular and large pore structures will significantly reduce the mechanical properties of foamed concrete.

**Figure 7:**
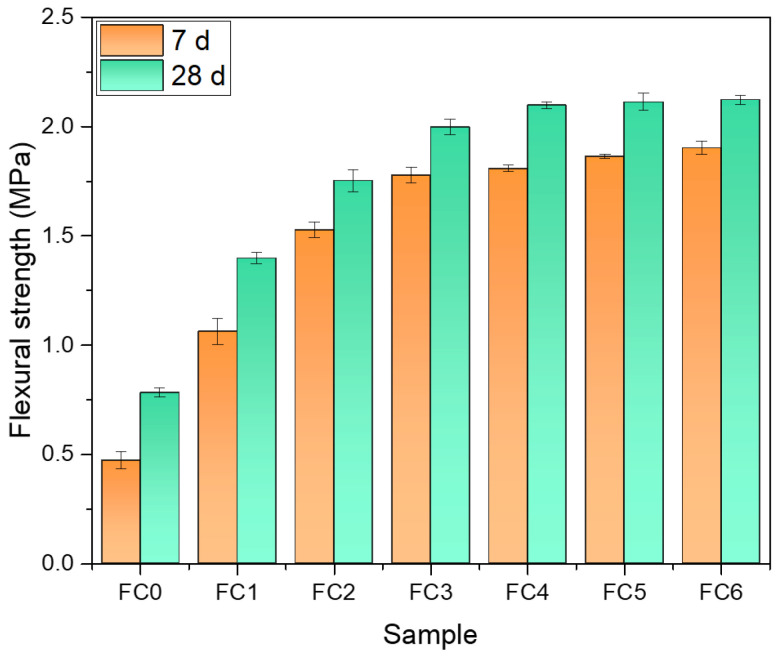
Flexural strength of specimens after curing for 7 d and 28 d.

**Figure 8:**
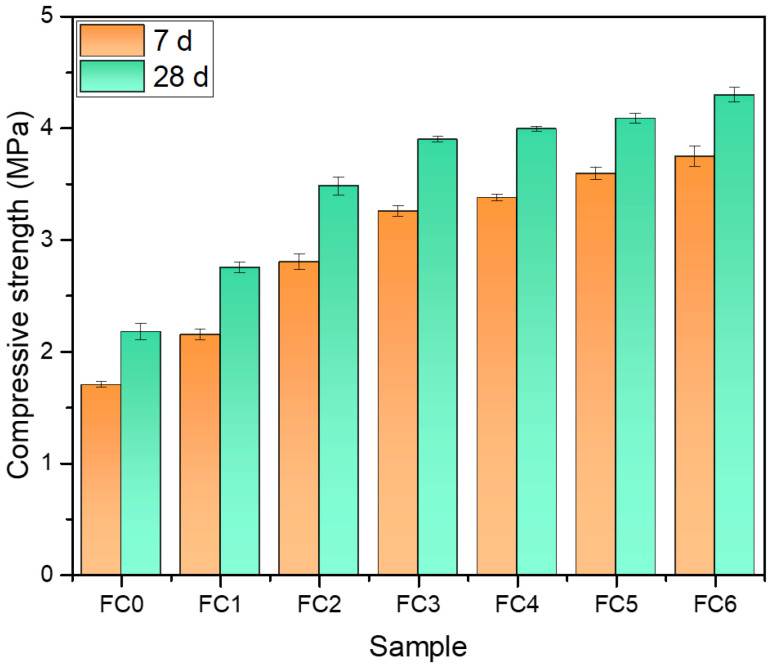
Compressive strength of specimens after curing for 7 d and 28 d.

Figure 7 and Figure 8 show the results of flexural and compressive strength tests of foam concrete specimens with different NA admixtures after curing 7 d and 28 d, respectively. It can be seen that the flexural strength of the specimens cured for 7 d and 28 d increased significantly with the increase in NA dosage before reaching a 0.18% dosage. After that, it rose slightly with the increase in NA doping. The flexural strength of FC3 is 1.78 MPa and 2.00 MPa at 7 d and 28 d, respectively, which is increased by 279% and 156% compared to FC0 (0.47 MPa at 7 d and 0.78 MPa at 28 d).

In Figure 8, the compressive strength of the specimens always has a large upward trend with the increase in NA doping, especially when the age of curing is 7 d. The compressive strength of FC0 is 1.70 MPa at 7 d and 2.18 MPa at 28 d. At 7 d, the compressive strength of FC3 and FC6 are 3.26 MPa and 3.75 MPa, respectively, an increase of 92% and 121% compared with that of FC0. At 28 d, the compressive strength of FC3 and FC6 are 3.90 MPa and 4.30 MPa, respectively, an increase of 79% and 97% in comparison with that of FC0. The results indicate that NA, as a foam stabilizer, is beneficial to the mechanical properties of foamed concrete.

Compared to previous research in which nano-materials were used as foam agents, the compressive strength of foamed concrete with 5% NA in the foam increased by 80% at 28 d [23], while that of foamed concrete with 8% nano-ettringite in the foam increased by 48% at 28 d [6]. Therefore, it can be concluded that the foam and foamed concrete prepared in this study demonstrated a superior strength enhancement.

#### 3.3.2. Micro Analysis

**Figure 9:**
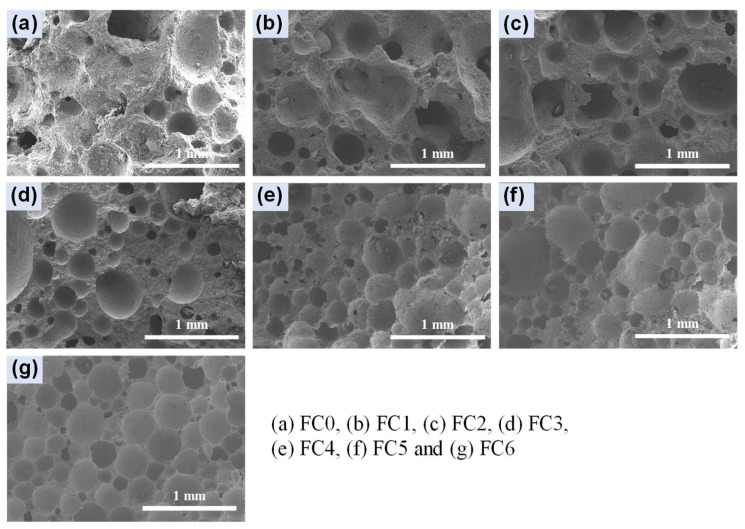
SEM images of specimens at 28 d.

**Figure 10:**
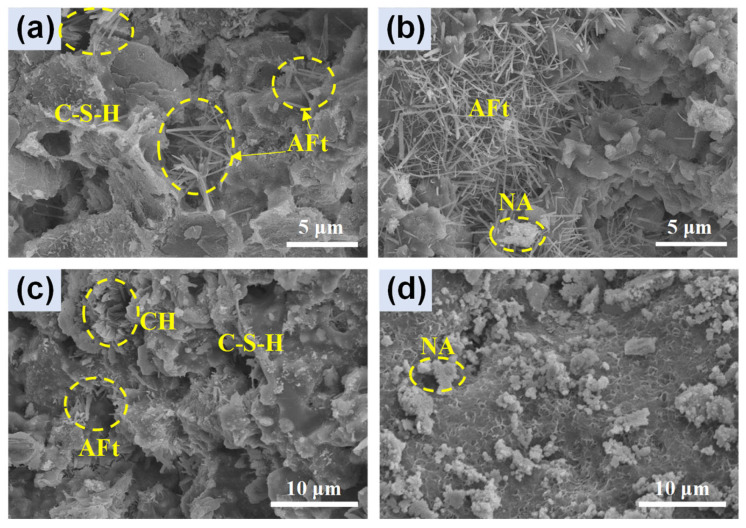
SEM images of specimens: (**a**) FC0 at 7 d, (**b**) FC6 at 7 d, (**c**) FC0 at 28 d, and (**d**) FC6 at 28 d.

Figure 9 and Figure 10 show the SEM images of each group of specimens at low and high magnification. As can be seen from Figure 9a, it is difficult for the foam to maintain a more complete spherical shape upon its incorporation into the cement when NA is not doped. The size of the pores varies greatly. This uneven distribution of pores will lead to an uneven distribution of stress in the foam concrete during the stressing process, and the specimen will be prone to cracking and then fracture at these large holes. With the increase in NA doping, the pores’ distribution becomes gradually homogeneous, and the difference in pore size decreases. In general, the closer the pore structure is to a sphere, the better the object’s resistance to compression for a constant volume [39]. This is one of the reasons why the compressive strength increases with the increase in NA doping.

The hydration products of the cement paste mainly consist of Ca(OH)_2_ and C-S-H gel, small amounts of AFm and AFt, and some unhydrated C_3_S and C_2_S minerals [40]. Figure 10 shows the SEM images of specimens with and without NA. After curing for 7 d, it can be seen that FC0 (Figure 10a) contains needle-like AFt and C-S-H gels, while FC6 (Figure 10b) exhibits a greater amount of AFt and some NA compared to FC0. After curing for 28 d, FC0 (Figure 10c) shows hydration products and pores on its surface, while FC6 (Figure 10d) presents a dense surface with some NA. This indicates that the addition of NA promotes the hydration of cement and results in a denser structure, further supporting the strength test results.

**Figure 11:**
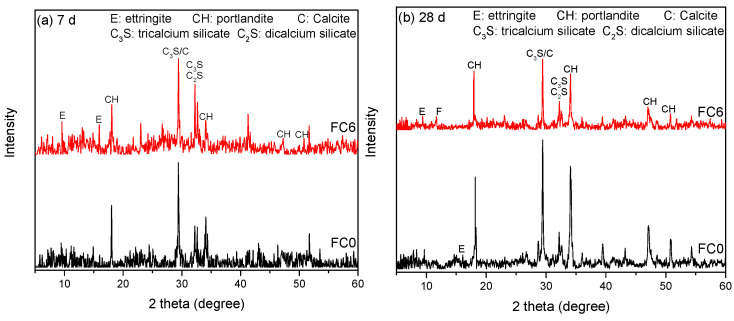
XRD pattern of specimens: (**a**) 7 d and (**b**) 28 d.

The XRD patterns of the foamed concrete after curing for 7 d and 28 d are shown in Figure 11. It can be seen that the addition of NA promotes the generation of AFt in the early stage of hydration. During the hydration process, the Ca(OH)_2_ generated by hydration is continuously consumed through reaction with NA [41].

A large amount of AFt was generated on the surface of the specimens after the addition of NA. NA dissolved in water initially forms Al(OH)^4−^. The dissolved Al(OH)^4−^ then reacts with the gypsum in cement to form AFt [42]. The consumption of gypsum further promotes the hydration of C_3_A. Therefore, the addition of NA particles not only accelerates the reaction of the silicate phase as the nucleus but also promotes the reaction of the aluminate phase and the formation of AFt. This increases the early strength of the foam concrete. NA has a large surface area and high activity, making it capable of undergoing pozzolanic reactions during a reaction [43]. It consumes the calcium hydroxide produced by hydration, generating products such as C-S-H and C-A-H [44]. This reaction fills the pores between the hydration products, resulting in a denser foam concrete microstructure.

Overall, due to the nucleation, chemical activity, and filling effect of NA in cementitious materials, the number of large voids in the cement matrix is reduced. This improvement is reflected in its macroscopic properties, significantly enhancing the mechanical properties of the foamed concrete.

## 4. Conclusions

In this study, a novel approach was introduced to enhance the stability of the foam in foamed concrete by incorporating NA into a foaming agent containing SDS and HPMC through physical foaming. This approach not only improved the stability of the foam but also increased the strength of the foamed concrete. The results were as follows:

1. NA effectively reduced the drainage volume and settlement distance of the foam. At a dosage of 0.18% NA, there was no bleeding within 20 min. The drainage volume decreased to 1.2 mL at 40 min and 2.4 mL at 60 min, while the settlement distance reduced to 0.1 cm, 0.4 cm, and 0.7 cm, respectively.
2. NA was distributed at the gas–liquid interface and within the liquid film, acting as a barrier. It increased the thickness of the liquid film and delayed the liquid discharge rate, helping to separate the adjacent bubbles and making bubble aggregation more difficult. This reduced the tendency for bubble aggregation, thereby enhancing foam stability.
3. Due to the stabilizing effect of NA on the foam, the precast foam formed a fine and uniform pore structure in the hardened foamed concrete. NA promoted the formation of early AFt, and its secondary hydration led to the continuous consumption of Ca(OH)_2_. This resulted in a more adequate hydration reaction, thereby enhancing the mechanical properties of the foamed concrete.
4. When the NA dosage was 0.36%, the 7 d and 28 d flexural strengths of the foamed concrete increased by 304% and 172%, respectively, and the compressive strengths increased by 152% and 96%, respectively, compared to those seen without NA.

This study proposed a new method to enhance the performance of foamed concrete, offering a reference for improving the foamed concrete used in engineering applications such as subgrade and thermal insulation buildings. However, this research focused only on the properties of foam and the short-term mechanical performance of foamed concrete, which may limit its practical application. Further research on the basic characteristics of foamed concrete containing NA is planned for the future from the perspective of practical applications, such as thermal conductivity, shrinkage, etc.

## Figures and Tables

**Table 1:** Properties of cement.

Properties	Values
Physical properties	
Density (g/cm^3^)	3.312
Specific surface area (m^2^/g)	0.34
Fineness (80 μm, %)	7.8
Initial setting time (min)	151
Final setting time (min)	243
3 d compressive strength (MPa)	24.5
28 d compressive strength (MPa)	53.5
Chemical components	
SiO_2_ (%)	22.71
Al_2_O_3_ (%)	5.64
CaO (%)	61.33
Fe_2_O_3_ (%)	3.38

**Table 2:** Mixed proportion of foaming agents (wt%).

Sample	SDS	HPMC	NA	Water
NA0	0.18	0.12	0	100
NA0.06	0.18	0.12	0.06	100
NA0.12	0.18	0.12	0.12	100
NA0.18	0.18	0.12	0.18	100
NA0.24	0.18	0.12	0.24	100
NA0.30	0.18	0.12	0.30	100
NA0.36	0.18	0.12	0.36	100

**Table 3:** Mixture proportions of foamed concrete.

Sample	Cement (g)	W/C	NSF (g)	Foam (g)
FC0	600	0.45	1.2	30 (NA0)
FC1	600	0.45	1.2	30 (NA0.06)
FC2	600	0.45	1.2	30 (NA0.12)
FC3	600	0.45	1.2	30 (NA0.18)
FC4	600	0.45	1.2	30 (NA0.24)
FC5	600	0.45	1.2	30 (NA0.30)
FC6	600	0.45	1.2	30 (NA0.36)

## Data Availability

The original contributions presented in the study are included in the article, further inquiries can be directed to the corresponding author.
